# Consensus-driven target product profiles for curative sickle cell disease gene therapies

**DOI:** 10.1016/j.omtm.2024.101287

**Published:** 2024-06-22

**Authors:** Daima Bukini, Julie Makani, Joseph McCune, Dennis Lee, Cathy Bansbach, Serena De Vita, Dominic Kemps, Elianna Amin, Jonathan Spector, John Tisdale

**Affiliations:** 1 Sickle Cell Disease Program, Muhimbili University of Health and Allied Sciences, Dar es Salaam 65001, Tanzania; 2 SickleInAfrica, Clinical Coordinating Center, Muhimbili University of Health and Allied Sciences, Dar es Salaam 65001, Tanzania; 3 Imperial College London, SW7 2AZ London, UK; 4 HIV Frontiers, Global Health Accelerator, Bill & Melinda Gates Foundation, Seattle, WA 98109, USA; 5 Chinacat Enterprises LLC, Gig Harbor, WA 98332, USA; 6 Translational Clinical Oncology, Biomedical Research, Novartis, Cambridge, MA 02139, USA; 7 HIV Cure Africa Acceleration Partnership, Sommartel, NW1 8DS London, UK; 8 Global Health, Biomedical Research, Novartis, Emeryville, CA 94608, USA; 9 Global Health, Biomedical Research, Novartis, Cambridge, MA 02139, USA; 10 Cellular and Molecular Therapeutics Branch, National Heart, Lung, and Blood Institute, Bethesda, MD 20814, USA

**Keywords:** sickle cell disease, gene therapy, Africa, target product profile, drug discovery

## Abstract

Therapeutic innovation to address sickle cell disease (SCD) is at a historical apex, characterized by a drug discovery, development, and commercialization landscape that includes potentially curative gene therapies. Given the wide geographic distribution of SCD, with a major presence in Africa, it is imperative that new medicines are designed to meet the specific needs of persons with SCD everywhere. Target product profiles (TPPs) detail the desired attributes of new medicines and serve as a guide for drug developers. To support research efforts for curative treatments for SCD, we mobilized a large multi-disciplinary expert group to generate consensus-driven TPPs for *ex vivo* and *in vivo* SCD gene therapies, utilizing a modified Delphi methodology supplemented with virtual workshops. The main findings are TPPs that describe 20 minimal and optimal criteria for novel gene therapy products in categories of scope (3 criteria), performance/safety (11 criteria), manufacturing (4 criteria), and administration (2 criteria). TPPs for *ex vivo* and *in vivo* products differed in some performance/safety criteria and all criteria pertaining to manufacturing and administration. These outputs will ideally support development of durable treatments that are safe, efficacious, and practical for persons with SCD in global settings.

## Graphical abstract

**Figure:**
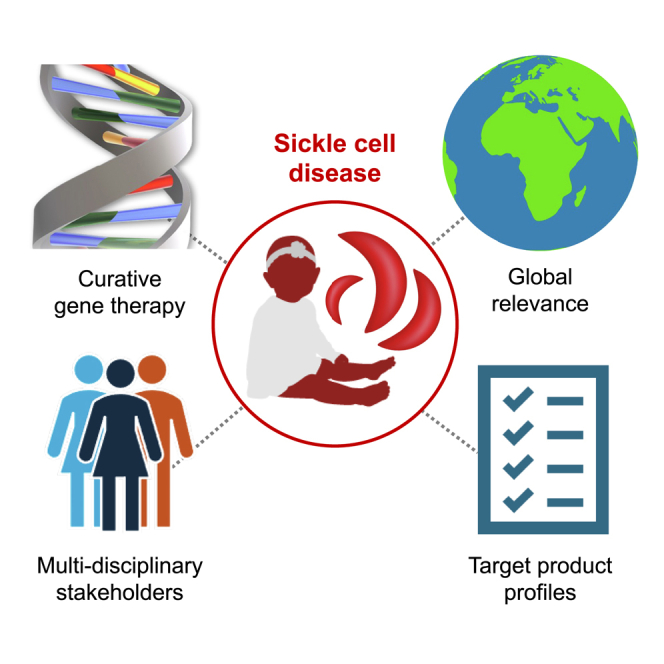

## Teaser

Bukini and colleagues share perspective on target product profiles developed through multi-stakeholder consensus to guide the design of *ex vivo* and *in vivo* gene therapies for sickle cell disease, with an emphasis on attributes to facilitate successful adoption by patient populations in global settings.

## Introduction

**Figure 1:**
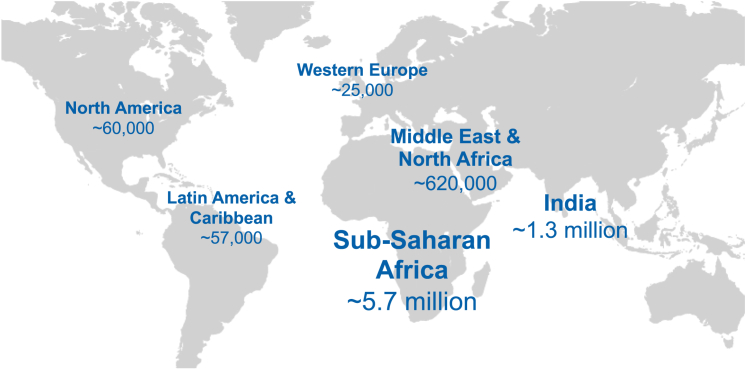
Estimates of the global distribution of sickle cell disease in regions where it is highly prevalent

The prevalence of sickle cell disease (SCD) is increasing, with nearly 8 million affected globally (Figure 1).^1^ All but approximately 5% of people with SCD live in low- and middle-income countries where reliable access to preventative healthcare and chronically administered disease-modifying therapies is often limited. Moreover, while currently available oral therapies can substantially mitigate the most severe clinical complications of SCD, some have attributes (such as the need for intensive monitoring) that preclude their use in certain populations. In addition, none of the available oral drugs are known to prevent the complications of SCD entirely.^2^ Thus, an alternative, safe, and cost-effective one-time curative therapy could offer important benefits for people living with SCD.

Although allogeneic hematopoietic stem cell (HSC) transplant can be curative, it is a complex process and carries the risk of chronic graft-versus-host disease and/or graft rejection in addition to other conditioning-related side effects. The procedure is further complicated by needs for a suitable stem cell donor and long-term post-transplant care. As such, less than 1% of people with SCD have been cured through transplant.^3^

Gene therapies are a relatively new class of therapeutics that can achieve durable effects by replacing, repairing, inactivating, or introducing genes into target cells. Genetic modulation is designed to take place *ex vivo* (“outside the body”) or *in vivo* (“inside the body”). For HSC-based gene therapies, a main difference between the two approaches is that the *ex vivo* approach is characterized by individualized HSC collection and manufacturing, along with needs for conditioning to prepare the bone marrow to receive the modified autologous stem cells; this contrasts with *in vivo* gene therapy whereby the process of genetic modulation of HSCs takes place inside the body without requirements for cell extraction or conditioning. There are currently more than 15 gene therapies for multiple diseases that are approved by the European Medicines Agency or the United States Food & Drug Administration (US FDA), with the first gene therapies to address SCD having been approved by the US FDA at the end of 2023.^4^^,^^5^

Target product profiles (TPPs) are tools used by drug developers to describe the desired characteristics of new medicines, e.g., details regarding the drug’s intended use, target population, safety and tolerability, clinical efficacy, route of administration, storage considerations, and other attributes. These profiles often describe both “minimum” (i.e., lowest acceptable level) and “optimum” (i.e., ideal target) performance scenarios, whereby drug candidates preferably meet all the minimum criteria and as many of the optimum targets as possible.

In recent years, several TPPs for new medicines and diagnostics have been generated by harnessing the collective input of a broad network of key stakeholders, including clinical specialists, community members, and those living with the disease in question.^6^^,^^7^^,^^8^^,^^9^ A main benefit of this methodology is to incorporate diverse perspectives in such a way as to increase the likelihood that the final product will be both adopted and used successfully for the purpose for which it was developed. For example, this approach helps to aggregate the views of drug developers (who are expert in technical characteristics of new therapies), health workers (who are expert in pragmatic aspects relating to administration of a new medicine in real world clinical settings), and, most importantly, persons living with SCD.

In the context of scientific advances that are enabling research and development of gene therapies for SCD, we sought to develop TPPs for SCD gene therapies through multi-stakeholder consensus with an emphasis on characteristics that will enable successful use in parts of the world where the condition is highly endemic. Theoretically, *in vivo* gene therapy is a more scalable solution compared with *ex vivo* products that require complex individualized manufacturing and, therefore, this approach would likely have particular advantages for reducing the burden of SCD in lower-resourced settings. However, despite the complexity of manufacturing, *ex vivo* gene therapies for SCD also hold the promise of delivering transformative impact for patients and, importantly, are more advanced in development and commercialization compared with *in vivo* gene therapies. Accordingly, this exercise focuses on both *ex vivo* and *in vivo* gene therapies for SCD.

## Multi-stakeholder consensus approach and findings

This study was a continuation of work initiated by the HIV Cure Africa Acceleration Partnership^10^ in late 2021 and took place in parallel with a similar exercise to determine a TPP for a cure for human immunodeficiency virus (HIV).^6^ The organizing group was comprised of employees at the Muhimbili University of Health & Allied Sciences in Tanzania (D.B. and Julie Makani), the Bill & Melinda Gates Foundation (Joseph McCune and D.L.), the Biomedical Research division of Novartis (S.D.V., E.A., and J.S.), the HIV Cure Africa Acceleration Partnership (D.K.), and United States National Institutes of Health (J.T.). The organizing group also included a professional consultant who specializes in the development of TPPs for therapeutics (C.B.).

Members of the organizing group, most of whom are engaged in research in the fields of SCD and/or gene therapies, began meeting in April 2022 with the stated purpose of helping to advance research and development for a safe and effective gene therapy cure for SCD intended for wide use in global settings by generating consensus-driven TPPs involving a network of multi-disciplinary stakeholders. A guiding principle was broad inclusivity with integral involvement of a community of representatives that live and work in settings where SCD is highly endemic.

The methodology of this study derives from established “Delphi”-type, consensus-based approaches designed to achieve convergence of opinion on a specific topic.^11^^,^^12^^,^^13^ The expert panel, referred to as “respondents” in this study, was envisioned to include a combination of persons with SCD, patient advocates, scientists, clinicians, policymakers, and other stakeholders concerned with the development of curative gene therapies for SCD. A list of approximately 100 prospective expert participants was developed by the organizing group based on existing professional networks and recommendations from recognized leaders in the field of SCD and/or gene therapies. In addition, the organizing group solicited potential expert participants through interaction with the Global Gene Therapy Initiative, an alliance of clinicians, scientists, engineers, advocates, patients, and others formed in 2020 with the goal of enabling access and implementation of curative gene therapies in low- and middle-income countries.^14^ A description of the planned study and invitation to participate was sent by email in September 2022 to prospective expert participants and those who wished to take part were included in the pool of respondents that received the electronic surveys.

**Figure 2:**
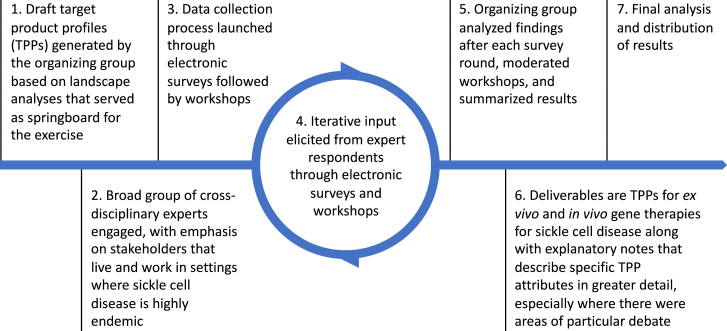
Methodology to generate target product profiles for gene therapy for sickle cell disease by multi-stakeholder consensus

The pre-determined methodology is summarized in Figure 2. Following a landscape analysis, draft TPPs for *ex vivo* and *in vivo* SCD gene therapies were developed by the organizing group to serve as the starting point for eliciting feedback from study respondents. The first electronic survey was distributed in October 2022 in which respondents were provided with background information about each TPP item and asked to answer a query about minimal and/or optimal scenario criteria using a pre-defined list of answers. Respondents could also write-in additional comments. A commercial survey software platform (Alchemer; www.alchemer.com) was used to distribute the electronic survey platform and to organize results for analysis by the organizing team.

After each electronic survey round, the organizing group reviewed the input in detail and assessed whether satisfactory consensus had been achieved. We defined a “majority” when more than 50% of respondents were in agreement and a “plurality” when a given measure was the favored choice by respondents but did not reach majority. To provide additional contextual detail regarding the findings, we used the term “great majority” when consensus was achieved by more than 75% of respondents.

For items that failed to reach at least a majority consensus, the organizing group studied the feedback provided by respondents and, if needed, re-framed the query relating to that item for the subsequent survey round to provide greater clarity for that item or to offer a different set of potential responses. It was planned from the outset that the rounds would continue until the organizing group determined that further rounds would be unlikely to significantly increase consensus on TPP items.

Following the electronic survey component of the study, optional virtual workshops were held to provide an opportunity for respondents to conduct open discussion on any of the TPP items, including those that had achieved consensus as well as those for which consensus was not achieved through electronic surveys. Recognizing the challenges in scheduling a meeting date and time that could accommodate such a large international group, dates for two workshops were offered and the same information was reviewed at each. The workshops were moderated by members of the organizing group.

Based on previous stakeholder consensus exercises, it was expected that consensus would be reached quickly on many of the items and that much of the effort would then be focused on achieving clarity on items that had not achieved consensus.^6^ It was anticipated that there would be three outputs of the study: (1) a TPP for an *ex vivo* gene therapy for SCD, (2) a TPP for an *in vivo* gene therapy for SCD, and (3) explanatory notes that reviewed the TPP items in detail and especially to describe aspects that generated debate among the respondents.

### Process and participants

The multi-stakeholder consultation took place between October 2022 and January 2023, characterized by systematic and iterative solicitation of input from an expert panel through the structured Delphi-type procedure described above.^12^^,^^13^ Two survey rounds were required to achieve at least a majority level of consensus for most TPP items, and two 1-h virtual workshops were subsequently held in April 2023. Questions administered through the survey rounds are provided in the supplemental information.

Ninety-seven prospective participants (including persons with SCD, patient advocates, physicians, scientists, and other stakeholders) were invited to participate, and a total of 56 respondents (58% of those solicited) contributed to some aspect of the exercise (listed in the acknowledgments). Fifty-five respondents participated in the survey consultation and 16 respondents participated in a workshop. The primary affiliation was an academic institution for approximately one-third of respondents; other affiliations included industry, government, and civil society (including persons with SCD and patient advocacy groups). More than 40% of respondents were living and working in Africa at the time of data collection and another 7% were nationals of India or Brazil, countries where SCD is also highly endemic.

The TPPs for *ex vivo* and *in vivo* gene therapies for SCD generated through this exercise are shown in Tables 1 and 2, respectively. Twenty items comprised the TPPs and were grouped into 4 categories: scope (3 items), performance/safety (11 items), manufacturing (4 items), and administration (2 items). There were differences between the TPPs for *ex vivo* and *in vivo* products in three items pertaining to performance/safety and all items pertaining to manufacturing and administration.

**Table 1:** Target product profile for *ex vivo* gene therapy for SCD

Characteristic	Minimal scenario	Optimal scenario
**Scope**		

Indication	persons with SCD caused by any homozygous β-globin chain abnormality including Hb SS, Hb SC, and other variants (e.g., thalassemia variants)	
Target population	persons with recurrent acute complications of SCD (e.g., vaso-occlusive crises, acute chest syndrome, etc.) aged ≥6 years	all persons with SCD regardless of severity including infants and young children
Special populations	safe and efficacious in non-pregnant, non-lactating women	safe and efficacious in women of reproductive age, including pregnant and lactating women with no risk to the developing fetus or breastfeeding child

**Performance/safety**		

Clinical efficacy	sustained (see “duration of benefit” below) improvement in biomarkers and >75% reduction in clinical complications of SCD and/or hospitalizations	normalization of biomarkers and durable (see “duration of benefit” below) prevention of clinical complications and hospitalizations
Duration of benefit	>10 years	lifetime
Treatment failure rate	<10%	<2%
Immunogenicity	clinically relevant immunogenicity acceptable if manageable in the outpatient setting	no clinically relevant immunogenicity observed
Drug-drug interactions	minimal drug-drug interactions with standard treatments for SCD, with any that arise being manageable in an outpatient setting	no drug-drug interactions
Impact on fertility	potential negative impact on fertility in genotypic females (e.g., reduced ovulation) or males (e.g., spermatogenesis dysfunction)	no impact on fertility
Conditioning	myeloablative conditioning	reduced-intensity or non-myeloablative conditioning
Potential for mutagenicity	very low	undetectable
Need for “on/off” switch	acceptable	not required
Vector shedding	allowable at a level that is acceptable by regulatory authorities	undetectable
Contraindications	pregnancy or complex co-morbidities including certain types of cancer, infectious diseases (e.g., HIV/AIDs, tuberculosis, hepatitis), autoimmune diseases, and end-organ disease (e.g., heart, kidney, lung diseases)	no contraindications

**Manufacturing**		

Formulation	infusible cell product	infusible cell product
Cell harvest	cells must be mobilized and obtained in a central facility that has specialized stem cell transplant capabilities	cells can be mobilized and harvested from individuals at peripheral level hospitals without specialized stem cell transplant capabilities
Cell manufacturing	a limited number of centralized manufacturing sites is acceptable provided they have capability to produce sufficient product quantities at a reasonable cost	many global manufacturing sites such that access is optimized
Stability and storage	frozen storage acceptable (i.e., requiring −20°C or colder)	storage and shipping at ambient temperature (i.e., room temperature) or refrigerated (i.e., at 2°C–8°C)

**Administration**		

Route of administration and selection criteria	single parenteral administration that may require additional medication(s) for *in vivo* selection of edited cells to improve efficacy	single parenteral administration without need for *in vivo* selection of edited cells to improve efficacy
Treatment location	inpatient setting	inpatient setting

**Table 2:** Target product profile for *in vivo* gene therapy for SCD

Characteristic	Minimal scenario	Optimal scenario
**Scope**		

Indication	persons with SCD caused by any homozygous β-globin chain abnormality including Hb SS, Hb SC, and other variants (e.g., thalassemia variants)	
Target population	persons with recurrent acute complications of SCD (e.g., vaso-occlusive crises, acute chest syndrome, etc.) aged ≥6 years	all persons with SCD regardless of severity including infants and young children
Special populations	safe and efficacious in non-pregnant, non-lactating women	safe and efficacious in women of reproductive age, including pregnant and lactating women with no risk to the developing fetus or breastfeeding child

**Performance/safety**		

Clinical efficacy	sustained (see “duration of benefit” below) improvement in biomarkers and >75% reduction in clinical complications of SCD and/or hospitalizations	normalization of biomarkers and durable (see “duration of benefit” below) prevention of clinical complications and hospitalizations
Duration of benefit	>5 years; >3 years if readministration is feasible	lifetime
Treatment failure rate	<10%; <20% if readministration is feasible	<2%
Immunogenicity	neutralizing activity against the vector or transgene or clinically relevant immunogenicity acceptable if manageable in the outpatient setting	no neutralizing activity or clinically relevant immunogenicity observed
Drug-drug interactions	minimal drug-drug interactions with standard treatments for SCD, with any that arise being manageable in an outpatient setting	no drug-drug interactions
Impact on fertility	potential negative impact on fertility in genotypic females (e.g., reduced ovulation) or males (e.g., spermatogenesis dysfunction)	no impact on fertility
Conditioning	not required	not required
Potential for mutagenicity	very low	undetectable
Need for “on/off” switch	acceptable	not required
Vector shedding	allowable at a level that is acceptable by regulatory authorities	undetectable
Contraindications	pregnancy or complex co-morbidities including certain types of cancer, infectious diseases (e.g., HIV/AIDs, tuberculosis, hepatitis), autoimmune diseases, end-organ disease (e.g., heart, kidney, lung diseases)	no contraindications

**Manufacturing**		

Formulation	injectable liquid or lyophilized product	injectable liquid or lyophilized product
Cell harvest	not required	not required
Cell manufacturing	not required	not required
Stability and storage	shelf life of at least 12 months; storage and shipping at −20°C or 2°C–8°C	shelf life >12 months; storage and shipping at room temperature

**Administrative**		

Route of administration and selection criteria	one or more parenteral administration(s) that may require additional medication(s) for *in vivo* selection of edited cells to improve efficacy	single parenteral administration without need for *in vivo* selection of edited cells to improve efficacy
treatment location	inpatient setting	safe for administration in an outpatient setting with no grade 3 (i.e., severe) or 4 (i.e., life-threatening) adverse events

### Findings: Scope

Items pertaining to scope were similar for the TPPs for *ex vivo* and *in vivo* products. The primary indication for SCD gene therapy is persons living with disease caused by any homozygous β-globin chain abnormality, including Hb SS, Hb SC, and other variants (e.g., thalassemia variants). Gene therapy is not indicated for sickle cell trait, which is characterized by a heterozygote state (i.e., a carrier state) in which only one gene is mutated and disease is not manifest.

A great majority (90%) of respondents felt that the target population should include individuals aged ≥6 years of age in the minimal scenario and persons of all ages, including infants and young children, in the optimal scenario. Given that children are significantly impacted by SCD, with clinical manifestations generally beginning to appear in infancy in parallel with the natural decline in fetal hemoglobin, it was recognized that treatment in the optimal scenario would take place in childhood before clinical complications arise. However, the respondents allowed for the minimal scenario, which excludes younger children if safety or efficacy issues of a novel gene therapy precluded its use in that population.

Sickle cell disease is known to increase the risk of maternal and fetal complications during pregnancy. If a safe and efficacious treatment is available, it may be ideal for women with SCD to be treated before pregnancy, especially in the case of an *ex vivo* therapy in which conditioning with chemotherapy agents is required. A great majority of respondents (>80%) felt that the minimal scenario would allow for the product to continue in development even if the product is associated with a risk of maternal, fetal, or infant toxicities in preclinical or clinical studies. Given there may be situations in which treatment during pregnancy or lactation is required, the optimal scenario envisions safe treatment in women who are pregnant or nursing.

### Findings: Performance/safety

Items relating to clinical efficacy, immunogenicity, drug-drug interactions, impact on fertility, potential for mutagenicity, need for an on/off switch, vector shedding, and contraindications were similar for the TPPs for *ex vivo* and *in vivo* products.

A majority (>70%) of respondents felt that the minimal scenario for clinical efficacy would be sustained improvement in biomarkers and >75% reduction in medical complications and/or hospitalizations related to SCD. Normalization of biomarkers and durable prevention of medical complications and hospitalizations would be expected in the optimal scenario (durability of clinical benefit is addressed below). The term “biomarker” in this context refers to a measurable biological substance indicating a pathogenic process associated with SCD, by example: markers of anemia (e.g., hemoglobin), hemoloysis (e.g., reticulocyte count, haptoglobin, lactase dehydrogenase), hemoglobin S polymerization (e.g., fetal hemoglobin levels), and inflammation (e.g., C-reactive protein and erythrocyte sedimentation rate).^15^ There are many potential medical complications associated with SCD that include but are not limited to vaso-occlusive pain crises, stroke, acute chest syndrome, and priapism.

It is possible that *in vivo* gene therapy vectors (e.g., viral vectors) or transgenes may elicit a neutralizing immune or inflammatory response in the recipient, which has implications for efficacy and/or safety. Similarly, *ex vivo* therapies could elicit undesired immune responses. In the optimal scenario, no neutralizing activity or clinically relevant immunogenicity would be observed following treatment. A great majority of respondents (nearly 90%) felt that neutralizing activity against the vector or transgene or clinically relevant immunogenicity would be acceptable in the minimal scenario if the condition would be manageable in an outpatient setting.

Persons with SCD are frequently treated with drugs that address the biologic basis of SCD (e.g., hydroxyurea), manage pain, prevent or treat infection, and/or treat other concurrent conditions. Interactions between these drugs and the gene therapy must be minimal or manageable in all cases. A great majority of respondents (>90%) felt that drug-drug interactions should be absent in the optimal scenario and infrequent in the minimal scenario, with any that arise being manageable in an outpatient setting.

As it relates to potential impact on fertility for recipients of a SCD gene therapy, the respondents felt that the optimal scenario would be characterized by no impact on fertility and that the minimal scenario could be associated with a potential negative impact on fertility in genotypic females (e.g., reduced ovulation) or males (e.g., spermatogenesis dysfunction). While gene transfer to germ cells is theoretically possible, the predominant concern on fertility is generally thought to be the impact of conditioning, which naturally makes this product attribute more relevant to *ex vivo* gene therapies. Depending on the characteristics of the final product, appropriate counseling regarding fertility risks would need to occur prior to therapy.

Insertional mutagenesis is a potential complication of gene therapy and a cause of genotoxicity that results from “off-target” insertion of a vector into the host DNA in such a way that disrupts normal cellular function and could potentially lead to complications such as leukemia or solid tumors (i.e., secondary malignancies).^16^ Ideally, the risk of insertional mutagenesis approaches zero; respondents agreed that the risk should therefore be undetectable in the optimal scenario and very low in the minimal scenario.

Additional safety concerns may be associated with *in vivo* gene therapy given its potential broad biodistribution, which could risk mutagenesis in a large number of varied cell types. This risk could theoretically be mitigated with increased targeting efficiency. Long-term follow-up of gene therapy recipients would presumably be needed in case mutagenesis is not detected in (shorter-term) clinical development programs.

There was poor consensus among the respondent group in the Round 1 survey for the items concerning an on/off switch and vector shedding. However, further analysis on these items by the organizing group revealed that the way in which the questions were initially posed to respondents may not have been sufficiently clear and that these technical aspects of gene therapy development may not have been familiar to many respondents. Questions relating to these items were re-engineered for the Round 2 survey and additional background information was included to better describe these concepts and their implications (see supplemental information). Both items then achieved a great majority (>80%) by respondents.

A gene therapy on/off switch is a gene that is inserted into modified cells that allows those cells to be eliminated (e.g., to “self-destruct”) should they become toxic or oncogenic. The ability to up- or downregulate the expression of the transgene may enhance safety. In the optimal scenario, the gene therapy would be sufficiently safe (i.e., without the risk for long-term toxicity or oncogenicity) such that an on/off switch is not needed. In the minimal scenario, it would be acceptable to include an on/off switch if needed to enhance the safety of the gene therapy product.

Viral vector shedding refers to the spreading of a viral vector (e.g., through saliva, urine, or excreta) from an individual into the environment after administration of gene therapy. Although gene therapies are relatively new types of medicine and the practical implications of shedding are uncertain, there are thought to be two main categories of potential risks: risk to the environment and risk of horizontal transmission to an untreated person. Health authorities in some countries require drug developers to provide information about viral vector shedding as part of their regulatory approval application. In addition, some approved gene therapy products recommend universal precautions (i.e., handwashing and safe handling of contaminated materials) by healthcare workers and family members for a limited period after administration to prevent the potential risk of horizontal transmission. To date, the risks of shedding following administration of gene therapies seem to be theoretical. Viral vector shedding has been observed in previous trials of gene therapies but there have not yet been (to the knowledge of the organizers of the current study) reports of negative outcomes that resulted from such shedding. Taking the above considerations into account, the respondents agreed that, in the optimal scenario, there will be undetectable shedding of vector and, in the minimal scenario, it is allowable for there to be vector shedding at a level that is considered acceptable by regulatory health authorities.

Contraindications represented the final performance/safety-related item that was similar between the *ex vivo* and *in vivo* TPPs. Given that the potential interactions of complex co-morbidities with gene therapy are not fully understood, the respondents felt that a gene therapy product would still be acceptable in the minimal scenario if its use was precluded in individuals with certain co-morbidities due to potential concerns relating to safety or efficacy. The list of such contraindicated conditions includes, but is not limited to, pregnancy, certain types of cancer, certain infectious diseases (e.g., HIV/AIDs, tuberculosis, hepatitis), autoimmune diseases, and end-organ disease (e.g., heart, kidney, lung diseases). In the optimal scenario, there would be no contraindications due to co-morbidities.

Three items pertaining to performance/safety differed between the TPPs for *ex vivo* and *in vivo* SCD gene therapy: duration of benefit, treatment failure rate, and conditioning. Duration of benefit and treatment failure rate were items that failed to achieve robust consensus through the Delphi surveys and required discussion at the workshops as well as among the organizing group prior to finalization.

In the optimal scenario, the gene therapy will result in a lifetime cure. However, it is possible that the efficacy of the gene therapy weakens over time. Given considerations such as the complexities associated with *ex vivo* gene therapy (e.g., requiring removal of patient cells, editing cells in a laboratory, autologous transplant of cells back to the patient, myeloablative therapy, and other procedures), cost, and potential complication risk (e.g., hematologic malignancies, exacerbated by the use of conditioning regimens), a majority (>50%) of respondents in the Delphi survey exercise felt that the minimum duration of benefit that should be targeted for *ex vivo* SCD gene therapy would be at least 10 years. However, some respondents suggested that a shorter duration of benefit (e.g., 3 or 5 years) would be sufficient, particularly if technological advances in the future led to reduced need for conditioning. It was also recognized through discussion in the workshops that drugmakers are likely to seek conditional approval based on less than 10 years of clinical trial data, with preclinical data supportive of prolonged durability of benefit.

For *in vivo* SCD gene therapy, the relative ease of administration (i.e., without the need for cell processing or patient conditioning) was felt to justify a shorter duration of benefit in the minimal scenario compared with an *ex vivo* product. While there was debate among respondents, there was plurality (involving nearly 40% of respondents) that a duration of benefit of at least 5 years would be appropriate in the minimal scenario; other perspectives favored a duration that was either shorter (e.g., 3 years) or longer (e.g., 10 years). Moreover, discussion in the workshops helped to conclude that the possibility of safe readministration of an *in vivo* product (should expression of the transgene wane) would make a duration of 1–3 years between treatments acceptable (noting that cost considerations would also need to be taken into account).

It is possible that, following successful engraftment, expression of the transgene may be lost. For current allogenic transplants in SCD, the failure rate is approximately 5%–10% and failures are typically observed within the first 100 days after administration.^17^^,^^18^ In the optimal scenario, the failure rate for a novel *ex vivo* SCD gene therapy will be less than 2%. In the minimal scenario, a higher treatment failure rate is tolerated and most respondents in the Delphi survey felt that the treatment failure rate should be <10% (while others felt the treatment failure rate could be as high as 20% or as low as 5%). For an *in vivo* product, a failure rate of <20% would be acceptable if it is possible to readminister the treatment.

A major benefit of *in vivo* therapy compared with *ex vivo* therapy is the lack of need for patient conditioning (e.g., myeloablative therapy). For *ex vivo* SCD gene therapy, standard myeloablative conditioning would be allowable in the minimal scenario and reduced-intensity or non-myeloablative conditioning would be preferred in the optimal scenario.

### Findings: Manufacturing

All items pertaining to manufacturing differed between *ex vivo* and *in vivo* products. These items attained a great majority (>80%) of consensus among respondents.

The formulation for *ex vivo* SCD gene therapy is an infusible cell product containing genetically modified forms of HSCs that were harvested from individuals. For an *in vivo* SCD gene therapy, currently available technologies suggest that the product would be delivered by intravenous infusion (e.g., provided in solution or lyophylized form); however, with technological advances in future years, it could perhaps be possible for the optimal scenario to be defined by intramuscular or subcutaneous injection.

For *ex vivo* SCD gene therapy, HSCs are expected to be harvested from peripheral blood after administration of an external agent that transiently mobilizes the cells from the bone marrow. In the optimal scenario, cells would be mobilized and harvested from individuals at most hospitals without the need for specialized stem cell transplant capabilities. The minimal scenario is defined by the need for mobilization and harvest to take place in a central facility that has specialized stem cell transplant capabilities. This procedure is not required for *in vivo* gene therapy; indeed, a major benefit of *in vivo* therapy compared with *ex vivo* therapy is the lack of need for cell harvesting and processing.

Manufacturing *ex vivo* gene therapy products is a complex and highly technical process. Key aspects include production of delivery vehicles (e.g., viral vectors) and cell processing. Centralized manufacturing has advantages for large-scale production but necessitates complex shipping logistics and cold chains to deliver products to treatment sites.^19^ As such, in the optimal scenario there would be many global manufacturing sites that serve to maximize access for persons with SCD. A limited number of centralized manufacturing sites would be acceptable in the minimal scenario, provided that they have capability to produce sufficient product quantities at a reasonable cost. As is the case with cell harvesting, cell manufacturing is not required for *in vivo* SCD gene therapy.

*Ex vivo* gene therapy products are produced for specific individuals and are delivered to the patient promptly after manufacturing; thus, shelf life may be of limited consideration for their use. The minimal scenario for frozen storage would be similar to conditions for currently approved *ex vivo* gene therapy products (i.e., requiring −20°C or colder). In the optimal scenario, storage and shipping would take place at ambient temperature (i.e., room temperature) or refrigeration (i.e., at 2°C–8°C) conditions.

Shelf life is a major consideration for an *in vivo* product since longer shelf lives in non-refrigerated or non-frozen conditions would be highly preferred to facilitate distribution in global settings, including parts of the world where SCD is highly endemic and characterized by tropical (i.e., hot and humid) climates. There was consensus among the respondents that the minimal scenario be defined by storage conditions that are similar to approved products (i.e., a shelf life of at least 12 months; storage and shipping at −20°C or 2°C–8°C). In the optimal scenario, the shelf life would be a year or longer with storage and shipping allowable at room temperature.

### Findings: Administration

Both items pertaining to administration differed between *ex vivo* and *in vivo* products. These items attained a great majority (>80%) of consensus among respondents.

*Ex vivo* and *in vivo* products would both be delivered by parenteral administration that may require additional medication(s) for *in vivo* selection of edited cells to improve efficacy (e.g., administration of an *in vivo* selection agent following treatment that enriches the population of edited HSCs to increase treatment effect). In the case of *ex vivo* SCD gene therapy, the product would be infused only once. For *in vivo* SCD gene therapy, the product may be administered multiple times depending on safety and efficacy; optimally, however, it is envisioned as a single-dose intravenous infusion over a specified time period (e.g., 60 min using a syringe pump).

For treatment location, approved *ex vivo* gene therapies currently require administration in an inpatient setting to monitor for potential severe adverse effects, and the minimal and optimal scenarios are currently defined by administration in an inpatient setting. However, it was acknowledged by the respondents that autologous transplantation is increasingly performed in an outpatient setting, and some centers are experimenting with conducting allogeneic transplant in an outpatient setting as well. Given that the risks of gene therapy should not be significantly different than for allogeneic transplant, outpatient administration for *ex vivo* SCD gene therapy could be the preferred optimal scenario in the future. For an *in vivo* SCD gene therapy product, administration in an inpatient setting would be acceptable in the minimal scenario; in the optimal scenario, however, the gene therapy product would be safe to administer in an outpatient setting, thereby maximizing accessibility.

### Discussion

While consensus-driven exercises have been previously conducted to devise recommendations for clinical management of SCD, to our knowledge this is the first multi-stakeholder consensus exercise to generate TPPs for SCD gene therapy (or, for that matter, TPPs for any SCD medicine).^20^^,^^21^^,^^22^ The approach leveraged established methodologies that have been applied to produce consensus-driven TPPs for therapeutics and diagnostics in other disease areas. Emanating from this exercise are TPPs for *ex vivo* and *in vivo* SCD gene therapies comprised of 20 attributes that commonly characterize TPPs for novel therapeutics.

Harnessing the collective perspective of a broad network of stakeholders was observed by the organizing group to have particular advantages: gene therapy as a therapeutic modality is a relatively novel technological advance and few individuals were seen to be technical “experts” in every item listed on the TPP. Thus, the aggregated input from a multi-disciplinary group, combining viewpoints from persons with SCD, physicians, scientists, and others, facilitated an analysis that was both balanced and discriminating. Most attributes of the TPP ultimately achieved robust consensus among the respondent group. For the few items that were determined by plurality rather than majority (e.g., duration of treatment benefit and treatment failure rate), the organizers worked to articulate minimal and optimal criteria that reflected the perspective of the greatest number of participants. It was also determined that those items should be qualified with an expanded description of the criteria (as provided in the sections above) to ensure an accurate representation that would be most helpful to drug developers and other users of the TPPs. Therefore, a faithful interpretation of the TPPs relies both on the TPP tables in this report as well as its explanatory notes.

Another important strength of this exercise was its intentional focus on global geographies, with an emphasis on regions where SCD is highly endemic, including Africa, India, and parts of South America. This manifested in the way certain TPP items were addressed (e.g., manufacturing, storage, and sites of administration) as well as in the makeup of the respondent group, as the organizers worked hard to ensure substantial representation from participants that live and work in those geographies.

In addition to the primary benefit of generating TPPs for SCD gene therapy, the organizing group appreciated a beneficial secondary gain of this exercise, namely: engagement of community in a discussion about gene therapy cures for SCD. The highly technical nature of early-stage drug discovery is such that the process can often be relatively insular and limited to academic or industrial researchers. In that context, the organizers found that the generation of TPPs through stakeholder consensus provided a natural opportunity to engage a large network during the period of drug discovery, a time when there would normally be little opportunity for wide interaction. This provided visibility into important considerations that shape early-stage drug discovery projects. In addition, the survey and workshop discussions provided an opportunity to help educate a community of SCD advocates on certain aspects of gene therapies that may not be intuitive to those lacking specialized technical training (e.g., concepts such as vector shedding and on/off switches). Finally, it was observed that collective action to generate TPPs helped to provide participants with a greater understanding of the drug discovery process; some respondents shared that they actually were not familiar with the concept of a TPP before the exercise started.

There are recognized limitations in the methodology and outputs of an exercise such as this. While an accurate response rate was challenging to ascertain (given that the organizers could not be sure that the invitation to participate was received by all the prospective participants to whom it was sent), higher numbers of respondents would have been welcomed, in particular from communities of persons with SCD and patient advocates. The TPPs generated through this activity are intended to be directional and not declarative; that is, they are the result of a cross-sectional exercise that represents the collective perspective of a group of stakeholders at one point in time. However, since many aspects of gene therapy science are rapidly evolving, the TPPs will ideally be “living documents” that are reviewed at regular intervals and adjusted as informed by progress in the field. Also, we acknowledge that some aspects of the TPP are challenging to put into practice; by example, data to support a 10-year duration of benefit are challenging to collect, despite the implementation of long-term follow-up protocols. In these instances, drug developers will need to work with regulators and other stakeholders to determine ways forward that meet the needs of persons with SCD while also being practical. Finally, we acknowledge that the TPP is aspirational in nature and that the evidence to support the feasibility of certain criteria will become available when clinical experiments are carried out. Ultimately, the TPP is intended to be a forward-looking “specification” for an impactful product based on best available current understanding of the field.

## Conclusions

A highly inclusive, multi-stakeholder effort defined the minimal and optimal attributes of *ex vivo* and *in vivo* gene therapies that aim to mitigate, and ideally cure, the physical manifestations of SCD, with an emphasis on characteristics that will enable widespread use of such therapies in global settings. More than 50 experts participated and contributed perspectives from the vantage point of persons with SCD, clinicians, scientists, and other disciplines. The outputs are intended to be used as guides by researchers, drugmakers, policymakers, and others that are seeking to discover and develop definitive novel therapeutics to address SCD.

## Supplemental information

Document S1. Tables S1 and S2

Document S2. Article plus supplemental information

## Footnotes

Supplemental information can be found online at https://doi.org/10.1016/j.omtm.2024.101287.

## References

[1] GBD 2021 Sickle Cell Disease Collaborators (2023). Global, regional, and national prevalence and mortality burden of sickle cell disease, 2000 – 2021: a systematic analysis from the Global Burden of Disease Study. Lancet Haematol..

[2] Tshilolo L., Tomlinson G., Williams T.N., Santos B., Olupot-Olupot P., Lane A., Aygun B., Stuber S.E., Latham T.S., McGann P.T. (2019). Hydroxyurea for Children with Sickle Cell Anemia in Sub-Saharan Africa. N. Engl. J. Med..

[3] sickle cell disease Commissioners Electronic address davidrees@kclacuk (2023). The Lancet Haematology Commission on sickle cell disease: key recommendations. Lancet Haematol..

[4] U.S. Food & Drug Administration Approved cellular and gene therapy products. https://www.fda.gov/vaccines-blood-biologics/cellular-gene-therapy-products/approved-cellular-and-gene-therapy-products.

[5] European Medicines Agency Approved Advanced Therapy Medicinal Products (ATMPs). https://www.ema.europa.eu/en/documents/report/cat-quarterly-highlights-approved-atmps-january-2023_en.pdf.

[6] Lewin S.R., Attoye T., Bansbach C., Doehle B., Dubé K., Dybul M., SenGupta D., Jiang A., Johnston R., Lamplough R. (2021). Multi-stakeholder consensus on a target product profile for an HIV cure. Lancet HIV.

[7] Vetter B., Beran D., Boulle P., Chua A., de la Tour R., Hattingh L., Perel P., Roglic G., Sampath R., Woodman M., Perone S.A. (2021). Development of a target product profile for a point-of-care cardiometabolic device. BMC Cardiovasc. Disord..

[8] Won K.Y., Gass K., Biamonte M., Dagne D.A., Ducker C., Hanna C., Hoerauf A., Lammie P.J., Njenga S.M., Noordin R. (2021). Diagnostics to support elimination of lymphatic filariasis-development of two target product profiles. PLoS Neglected Trop. Dis..

[9] Alonso-Padillaid J., Abril M., de Noya B.A., Almeida I.C., Angheben A., Jorge T.A., Chatelain E., Esteva M., Gascon J., Grijalva M.J. (2020). Target product profile for a test for the early assessment of treatment efficacy in chagas disease patients: An expert consensus. PLoS Neglected Trop. Dis..

[10] Dybul M., Attoye T., Baptiste S., Cherutich P., Dabis F., Deeks S.G., Dieffenbach C., Doehle B., Goodenow M.M., Jiang A. (2021). The case for an HIV cure and how to get there. Lancet HIV.

[11] Hsu C.C., Sandford B.A. (2007). The Delphi technique: making sense of consensus. Practical Assess. Res. Eval..

[12] Fink A., Kosecoff J., Chassin M., Brook R.H. (1984). Consensus methods: characteristics and guidelines for use. Am. J. Publ. Health.

[13] Linstone H., Turoff M. (1975).

[14] Adair J.E., Androski L., Bayigga L., Bazira D., Brandon E., Dee L., Deeks S., Draz M., Dubé K., Dybul M. (2023). Towards access for all: 1st Working Group Report for the Global Gene Therapy Initiative (GGTI). Gene Ther..

[15] Rees D.C., Gibson J.S. (2012). Biomarkers in sickle cell disease. Br. J. Haematol..

[16] High K.A., Roncarolo M.G. (2019). Gene therapy. N. Engl. J. Med..

[17] Abraham A., Hsieh M., Eapen M., Fitzhugh C., Carreras J., Keesler D., Guilcher G., Kamani N., Walters M.C., Boelens J.J. (2017). Relationship between mixed donor–recipient chimerism and disease recurrence after hematopoietic cell transplantation for sickle cell disease. Biol. Blood Marrow Transplant..

[18] Eapen M., Brazauskas R., Walters M.C., Bernaudin F., Bo-Subait K., Fitzhugh C.D., Hankins J.S., Kanter J., Meerpohl J.J., Bolaños-Meade J. (2019). Effect of donor type and conditioning regimen intensity on allogeneic transplantation outcomes in patients with sickle cell disease: a retrospective multicentre, cohort study. Lancet Haematol..

[19] Doxzen K., Cornetta K., Hongeng S., Kityo C., Mahlangu J., Makani J., Mathews V. (2022).

[20] Brandow A.M., Carroll C.P., Creary S., Edwards-Elliott R., Glassberg J., Hurley R.W., Kutlar A., Seisa M., Stinson J., Strouse J.J. (2020). Americal Society of Hematology 2020 guidelines for sickle cell disease: management of acute and chronic pain. Blood Adv..

[21] Sobota A.E., Shah N., Mack J.W. (2017). Development of quality indicators for transition from pediatric to adult care in sickle cell disease: A modified Delphi survey of adult providers. Pediatr. Blood Cancer.

[22] Yawn B.P., Buchanan G.R., Afenyi-Annan A.N., Ballas S.K., Hassell K.L., James A.H., Jordan L., Lanzkron S.M., Lottenberg R., Savage W.J. (2014). Management of sickle cell disease: summary of the 2014 evidence-based report by expert panel members. J. Am. Med. Assoc..

