# Supplementary material for: Consensus-driven target product profiles for curative sickle cell disease gene therapies
Source: Mol Ther Methods Clin Dev. 2024 Jun 22;32(3):101287. doi: 10.1016/j.omtm.2024.101287 (PMC11298580; doi:10.1016/j.omtm.2024.101287)
Supplement: Document S1. Tables S1 and S2 [file mmc1.pdf]

OMTM, Volume *32*

Supplemental information

Consensus-driven target product profiles for curative sickle cell disease gene therapies

Daima Bukini, Julie Makani, Joseph McCune, Dennis Lee, Cathy Bansbach, Serena De Vita, Dominic Kemps, Elianna Amin, Jonathan Spector, and John Tisdale

Table S1. Delphi survey round 1 technical questions.

Question number	Question
1	In the minimum scenario, would it be acceptable for the target population to include pediatric patients >6 years of age?
2	In the minimum scenario, must the gene therapy be safe and effective in infants and toddlers (i.e., children less than 6 years of age)?
3	In the minimum scenario, must the gene therapy be safe and effective in pregnant and nursing women?
4	In the minimum scenario, would the gene therapy be acceptable if it was only used in persons with severe disease, as evidenced by recurrent acute complications of SCD (e.g., vaso- occlusive crises, acute chest syndrome, etc.)?
5	In the optimum scenario there will be normalization of biomarkers (e.g., degree of anemia, markers of hemolysis). In the minimum scenario, would sustained improvement in biomarkers be sufficient?
6	A key outcome of the gene therapy will be reduction in medical complications and/or hospitalizations. In the optimum scenario, the gene therapy would prevent complications of SCD (e.g. vaso-occlusive crisis, acute chest syndrome, stroke, etc) and hospitalizations. In the minimum scenario, what degree of reduction in medical complications and/or hospitalizations should be targeted?
7	In the optimum scenario, the gene therapy will result in a lifetime cure. However, given the practical considerations associated with conducting long-term clinical trials, there may be limitations to how long the duration of benefit can be measured in a clinical trial. What is the minimum duration of benefit that should be targeted to have a significant clinical impact?
8	In the optimum scenario, the treatment failure rate will be low: less than 2% per year. In the minimum scenario, a higher treatment failure rate may be tolerated. What relapse rate is minimally acceptable for the in vivo approach?
9	Ex vivo gene therapy requires more technical resources to deliver compared to in vivo therapy, and therefore there could be differences in the accepted treatment failure rate. What relapse rate would be minimally acceptable for ex vivo gene therapy?
10	In the optimum scenario, the gene therapy will be administered in an outpatient setting without the need for special monitoring (i.e. without any severe adverse events). In the minimum scenario, would it be acceptable for the gene therapy to be administered in an outpatient setting even if it causes a low frequency of manageable/reversible adverse events that are Grade 3 (e.g., severe events that require hospitalizations or substantial intervention) or Grade 4 (e.g., life threatening events such as cardiovascular failure or need for intensive care)?
11	In the optimum scenario there will be no neutralizing or clinically concerning immunogenicity. In the minimum scenario, would it be acceptable for the gene therapy to be associated with immunogenicity provided that the condition is manageable in an outpatient setting?
12	"Viral vector shedding" refers to spreading of viral vector from a patient into the environment (e.g., through saliva, urine, or excreta). In the optimum scenario there will be no shedding of vector. In the minimum scenario, would it be acceptable for there to be limited shedding of the vector?

13	In the minimum scenario, would a “on/off switch” be needed? (i.e., a gene that is inserted into modified cells that allows modified cells to be controlled/self-destruct in case they become toxic or oncogenic)?
14	In the optimum scenario, the gene therapy will be delivered as a single intravenous injection with no need to subsequently select for edited cells to improve efficacy (“selecting” for edited cells would enrich the population of hematopoietic stem cells with edited cells). In the minimum scenario, would it be acceptable if additional medication for in vivo selection of edited cells is required?
15	In the optimum scenario there will be no drug-drug interactions between the gene therapy and other standard medicines that are administered to treat patients with SCD (e.g., hydroxyurea, pain medicines, etc). In the minimum scenario, would it be acceptable if there are minimal interactions that can be managed in an outpatient setting?
16	In the optimum scenario, patient cells can be harvested (i.e., obtained) in hospitals that do not have specialized stem cell transplant capabilities (e.g., capabilities such as exchange blood transfusion and hematopoietic stem cell transplant). In the minimum scenario, would it be acceptable if cells need to be harvested in a central specialized facility that has existing stem cell transplant capabilities?
17	The optimum target for efficiency of ex vivo genetic manipulation of hematopoietic stem cells is >75%. In the minimum scenario, what is the acceptable target?
18	In the optimum scenario there will be many global manufacturing sites. In the minimum scenario, would a limited number of global manufacturing sites be acceptable, provided that they had the capability to rapidly scale-up production at cost/dose in such a way as to enable broad use?
19	In the optimum scenario, the cell product will be able to be stored at ambient (i.e., room temperature) or refrigerated temperatures. In the minimum scenario, would it be acceptable for the cell product to be frozen (i.e., -20oC or higher)?
20	In the optimum scenario the gene therapy product will have a shelf life of >36 months at ambient temperature. In the minimum scenario, would it be acceptable to have a shelf life of 6 months?
21	In the optimum scenario, the cell product will be able to be stored at climactic zone 4b (i.e., hot and humid temperatures) with heat stability demonstrated to 40oC for 1 week. In the minimum scenario, would it be acceptable for the cell product to be frozen (i.e., -20oC or higher)?
22	Please feel free to write any comments in the space below. Please feel free to provide feedback on the process of taking the survey or share any further insights regarding any of the specific survey questions.

Table S2. Delphi survey round 2 technical questions.

**Question number**	**Question**
1	*Target population.* It is well recognized that in some parts of the world morbidity and mortality in sickle cell disease are particularly high in childhood. In the optimum scenario, the gene therapy will be safe and effective in patients of any age, including infants and toddlers (i.e., children less than 6 years of age). However, it is possible that a new gene therapy could only be used safely and effectively in patients > 6 years of age. Would it still be acceptable to proceed

	with development of a gene therapy if it could only be used in patients > 6 years of age?
2	*Storage of an in vivo gene therapy*. There are only a few approved in vivo gene therapies for any indication. These therapies typically require storage at a frozen temperature (36 months at ambient temperatures (i.e., room temperature). However, that may be challenging to achieve given the current technologies that are available to drug developers. Therefore, would it be acceptable in the minimum scenario for a novel in vivo gene therapy to require similar storage conditions to approved products—specifically, stable for at least one year (12 months) when stored at a frozen temperature
3	*Duration of benefit for an ex vivo gene therapy.* In the optimum scenario, the gene therapy will result in a lifetime cure. However, it is possible that the efficacy of the ex vivo gene therapy weakens over time. What is the minimum duration of benefit that should be targeted for an ex vivo gene therapy product to have significant clinical impact? (Please recall that ex vivo gene therapy typically requires removal of patient cells, editing cells in a laboratory, autologous transplant of cells back to the patient, myeloablative therapy, and other procedures).
4	*Duration of benefit for an in vivo gene therapy*. In the optimum scenario, the gene therapy will result in a lifetime cure. However, it is possible that the efficacy of the in vivo gene therapy weakens over time. What is the minimum duration of benefit that should be targeted for an in vivo gene therapy product to have significant clinical impact? (Please recall that in vivo gene therapy typically requires one or several injections without the need for removal of patient cells from the body).
5	*Treatment failure for an ex vivo gene therapy.* It is possible that a gene therapy product will fail to be effective in a patient after it is administered. For current allogenic transplants in sickle cell disease, the failure rate is approximately 5-10% and failures are typically observed within the first 100 days or so after administration. In the optimum scenario, the failure rate for a novel ex vivo sickle cell disease gene therapy will be less than 2%. In the minimum scenario, a higher treatment failure rate may be tolerated. What failure rate would be acceptable for an ex vivo gene therapy in the minimum scenario?
6	*Treatment failure for an in vivo gene therapy.* It is possible that a gene therapy product will fail to be effective in a patient after it is administered. For current allogenic transplants in sickle cell disease, the failure rate is approximately 5-10% and failures are typically observed within the first 100 days or so after administration. In the optimum scenario, the failure rate for a novel in vivo sickle cell disease gene therapy will be less than 2%. In the minimum scenario, a higher treatment failure rate may be tolerated. What failure rate would be acceptable for an in vivo gene therapy in the minimum scenario?
7	*Treatment setting for an in vivo gene therapy.* Ex vivo gene therapy for any indication requires administration in an inpatient setting to monitor for potential severe adverse effects. For an in vivo gene therapy for sickle cell disease, the optimum scenario is for the gene therapy to be administered in an outpatient setting with limited needs for special monitoring. In the minimum scenario, would it be acceptable for an in vivo gene therapy to be delivered in an inpatient facility in case that would be needed to monitor for potential severe adverse effects?
8	*Vector shedding.* “Viral vector shedding” refers to spreading of viral vector from a patient into the environment (e.g., through saliva, urine, or excreta) after administration of gene therapy. Because gene therapies are still relatively new types of medicines, the practical implications of shedding are uncertain. There are

	thought to be two main categories of potential risks: risk to the environment and risk of horizontal transmission to an untreated individual. Health authorities in some countries require drug developers to provide information about viral vector shedding as part of their regulatory approval application. In addition, some approved gene therapy products recommend universal precautions (i.e., handwashing and safe handling of contaminated materials) by healthcare workers and family members for a limited period after administration to prevent the potential risk of horizontal transmission. Note that, to date, the risks of shedding are theoretical. Viral vector shedding has been observed in previous trials of gene therapies but there have not yet been (to our knowledge) any reports of negative outcomes that have resulted from shedding.In the optimum scenario there will be no shedding of vector. In the minimum scenario, would it be allowable for there to be vector shedding at a level that is considered acceptable by regulatory health authorities?
9	*On/off switch.* A gene therapy "on/off switch" is a gene that is inserted into modified cells that allows modified cells to be controlled (e.g., "self-destruct") in case they become toxic or oncogenic. In the optimum scenario, the gene therapy will be sufficiently safe (i.e., without the risk for long- term toxicity or oncogenicity) such that an of/off switch is not needed. In the minimum scenario, would it be acceptable to include an on/off switch if needed to enhance the safety of the gene therapy product?
10	Please feel free to write any comments in the space below. Please feel free to provide feedback on the process of taking the survey or share any further insights regarding any of the specific survey questions.
